# Supplementary material for: Mechano-inhibition of endocytosis sensitizes cancer cells to Fas-induced Apoptosis
Source: Cell Death Dis. 2024 Jun 22;15(6):440. doi: 10.1038/s41419-024-06822-3 (PMC11193792; doi:10.1038/s41419-024-06822-3)
Supplement: Supplementary file 1 — Supplementary Information [file 41419_2024_6822_MOESM1_ESM.pdf]

Mechano-inhibition of Endocytosis Sensitizes Cancer Cells to Fas-induced Apoptosis

Mehmet H. Kural[1,2,*,†], Umidahan Djakbarova[3,*], Bilal Cakir[4], Yoshiaki Tanaka[4,5], Emily T. Chan[3,6], Valeria Arteaga Muniz[3], Yasaman Madraki[3], Hong Qian[1,2], Jinkyu Park[7], Lorenzo R. Sewanan[8], In-Hyun Park[4], Laura E. Niklason[1,2,†], Comert Kural [3,6,†]

Supplementary Information

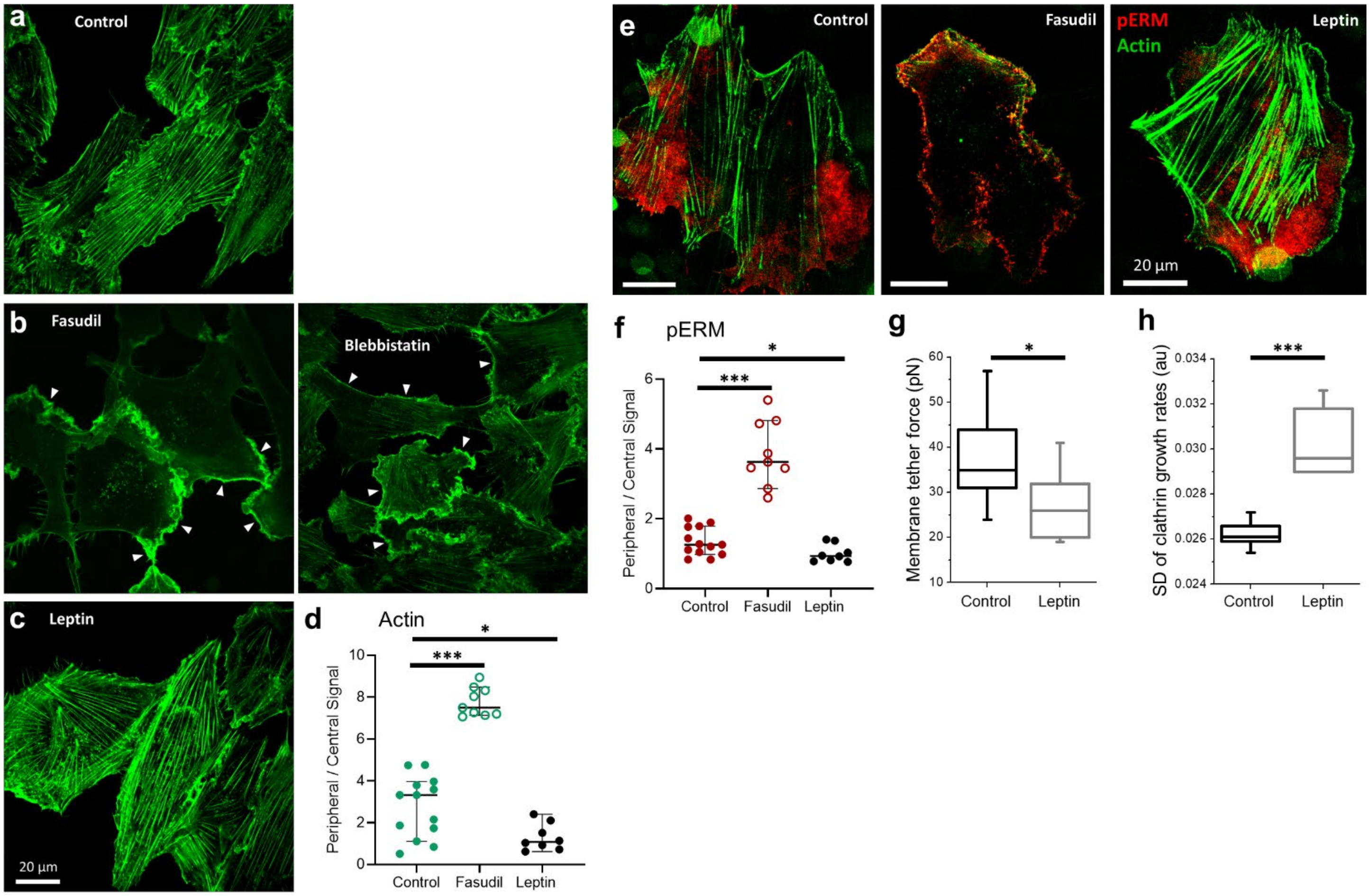

Supplementary Figure 1. a-c, Immunofluorescence microscopy shows the distribution of actin in SUM159 cells under different conditions. **b**, Reduction of actomyosin contractility by treatment with fasudil or blebbistatin resulted in the disappearance of actin stress fibers observed in untreated cells (a) and the accumulation of actin signal in the cell cortex, as marked by arrowheads. **c**, Activation of Rho-kinase by leptin treatment increased the formation of actin stress fibers. **d,** Quantification of the actin signal in the peripheral regions of the cell versus the center demonstrates that fasudil treatment causes a greater than two-fold increase in actin deposition in the cell cortex. Leptin treatment, on the other hand, reduces this ratio significantly. **e-f**, Reduction of actomyosin contractility by fasudil treatment resulted in the peripheral distribution of phosphorylated ERM (pERM) protein (red) signal along with actin (green). **g,** Membrane tether forces quantified by optical tweezers experiments significantly reduced upon leptin treatment. **h,** Reduced membrane tension by leptin treatment gave rise to significantly higher clathrin-mediated endocytosis dynamics. ***$p<0.001$, *$p<0.05$; two-tailed t test. Scale bar, 20 µm.

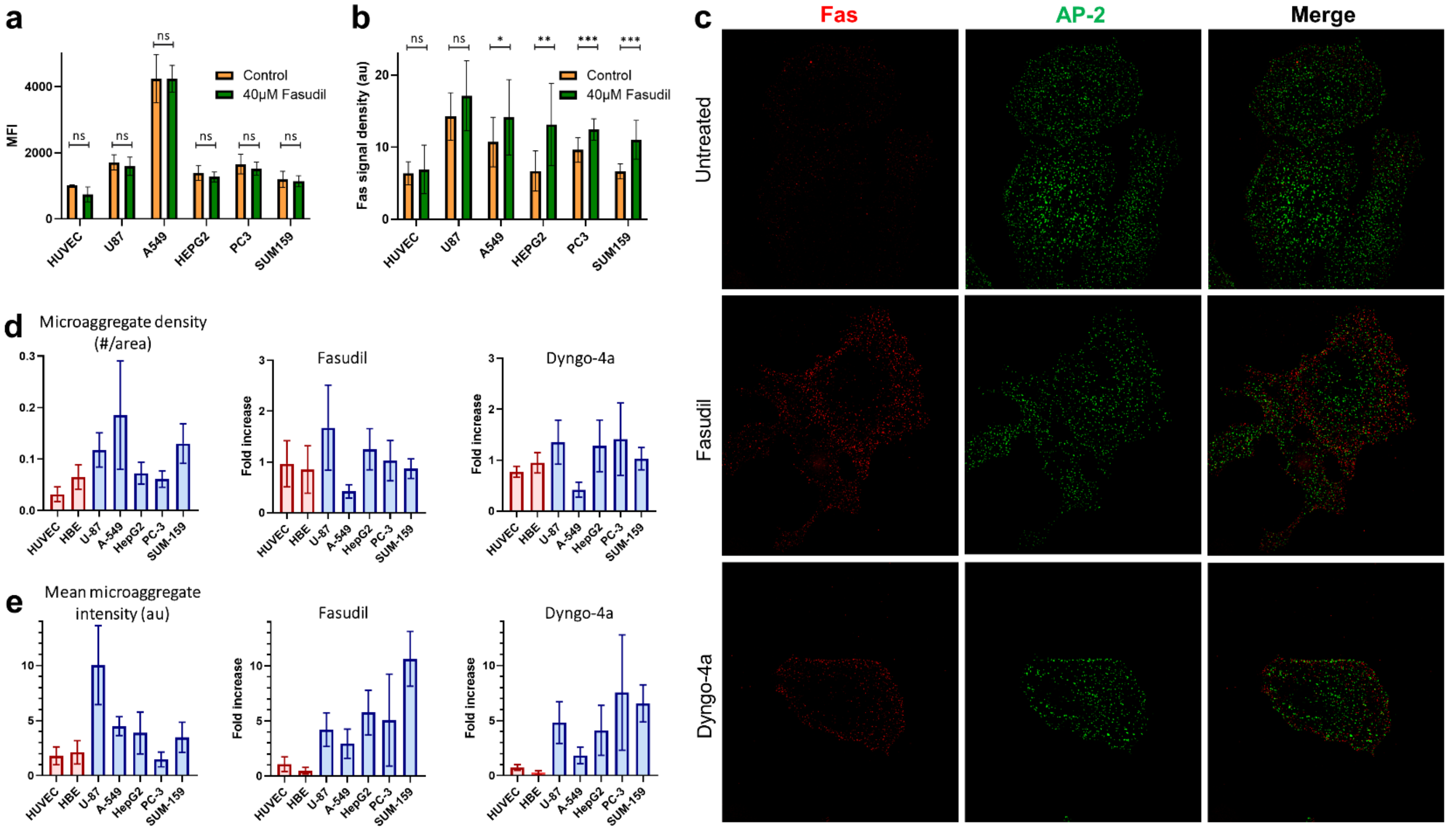

Supplementary Figure 2. a, Flow cytometry analyses were conducted to test the effect of 40 µM fasudil on the total expression levels of Fas in HUVEC, U87, A549, HepG2, PC3 and SUM159 cells. **b,** Surface expression of Fas was evaluated in the same cell types through immunofluorescence labeling while employing cold block. **c,** Spinning disk confocal fluorescence microscopy was used to image Fas microaggregates exclusively at the ventral/bottom surface of SUM159 cells that were genome edited to express AP2-EGFP, a marker of endocytic clathrin coats at the plasma membrane. The AP2-EGFP signal was used to verify that the image plane coincides with the ventral surface of the plasma membrane in these assays. Scale bar, 20 µm. **d,** The density of Fas microaggregates (mean ± sd) is shown for different cells (left). Treatment with 40 µM fasudil (middle) or 5 µM dyngo-4a (right) for 2 hours did not significantly increase microaggregate density in any of the cell types. **e**, Mean fluorescence intensity of Fas microaggregates for different cells (left). Treatment with 40 µM fasudil (middle) or 5 µM dyngo-4a (right) for 2 hours did not increase the intensity of Fas microaggregates in HUVECs or HBE cells but did substantially increase this intensity in cancer cells (U87, A549, HepG2, PC3 and SUM159 cells). The results are shown as mean fluorescence intensity +/- standard deviation (ns: nonsignificant, *** $p<0.001$, ** $p<0.01$, and * $p<0.05$; t test).

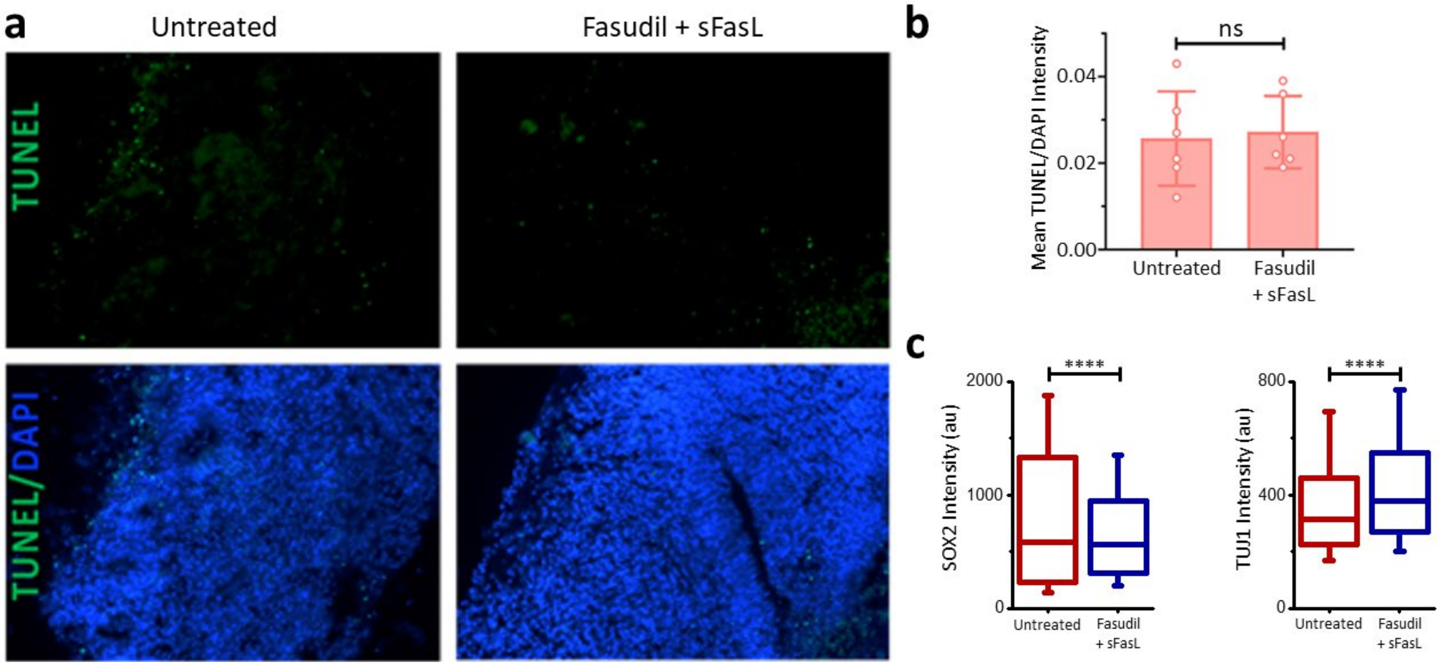

Supplementary Figure 3. a, Seventy-eight-day-old healthy organoids were treated without or with both fasudil and sFasL. **b,** The ratio of TUNEL+ cells to the total number of nuclei was not significantly different in the combination treatment group and the untreated group ($p = 0.79$; unpaired t test). **c,** Box plots show the SOX2 and TUJ1 fluorescence staining levels in organoid cells before and after fasudil+sFasL treatment ($N_{CellsBefore} = 1086$; $N_{CellsAfter} = 1506$). We detected ~13% increase in TUJ1 intensity (391.4 ± 9.0 vs 440.9 ± 6.1, mean ± sem) and ~20% decrease in SOX2 intensity (833.3 ± 21.7 vs 695.8 ± 12.9, mean ± sem) upon treatment.

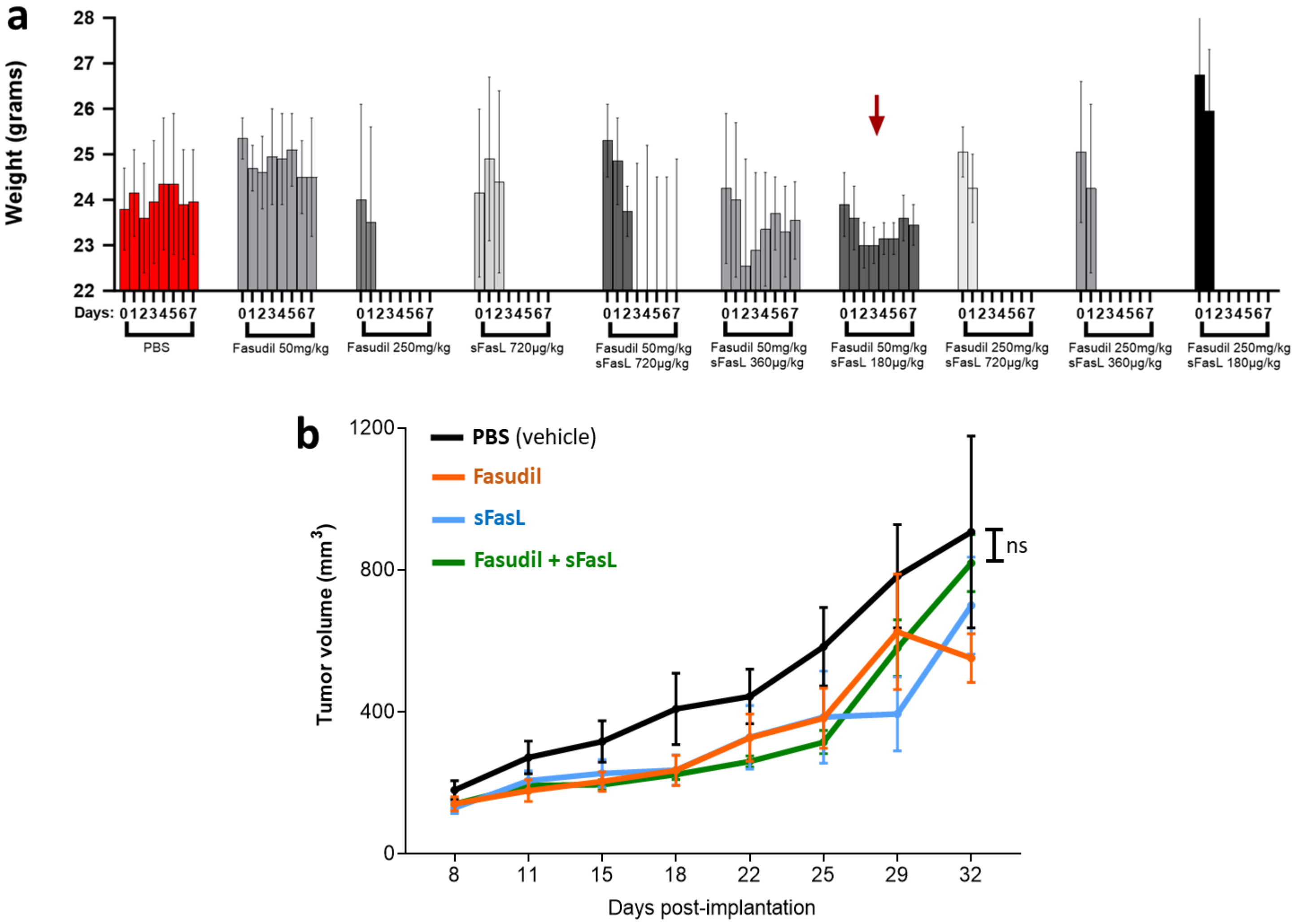

Supplementary Figure 4. a, Results of the maximum tolerated dose assays show the weight of mice in different days of the intraperitoneal delivery of fasudil and/or sFasL at different concentrations. The doses used in our xenograft assays (50mg/kg fasudil and 180µg/kg sFasL) are marked with the red arrow. **b,** Intraperitoneal delivery of different treatments did not result in a significant difference in tumor volume (Ordinary one-way ANOVA). Overall, the fasudil+sFasL combination performed better than PBS, but the difference was not statistically significant.

Supplementary Videos:

Video 1: Z-stack acquired by spinning disk confocal imaging shows the surface localization of Fas (red) in a SUM159 cell genome edited to express AP2-EGFP (green).

Video 2: Untreated iPS-cardiomyocyte monolayer that is spontaneously contracting on the tissue culture plastic.

Video 3: 48 hours of treatment with combinations of fasudil - sFasL combination did not affect the contraction ability of iPS-cardiomyocyte monolayer.
